# Blood–Brain Barrier Disruption and Hemorrhagic Transformation in Acute Ischemic Stroke: Systematic Review and Meta-Analysis

**DOI:** 10.3389/fneur.2020.594613

**Published:** 2021-01-21

**Authors:** Francesco Arba, Chiara Rinaldi, Danilo Caimano, Federica Vit, Giorgio Busto, Enrico Fainardi

**Affiliations:** ^1^Stroke Unit, AOU Careggi, Florence, Italy; ^2^NEUROFARBA Department, University of Florence, Florence, Italy; ^3^Neuroradiology, AOU Careggi, Florence, Italy; ^4^Department of Experimental and Clinical Medicine, University of Florence, Florence, Italy

**Keywords:** blood-brain-barrier, ischemic stroke, hemorrhagic transformation (HT), perfusion tomography, magnetic resoance imaging, intravenous thrombolysis, endovascular treatment (EVT)

## Abstract

**Introduction:** Hemorrhagic transformation (HT) is a complication of reperfusion therapy for acute ischemic stroke. Blood–brain barrier (BBB) disruption is a crucial step toward HT; however, in clinical studies, there is still uncertainty about this relation. Hence, we conducted a systematic review and meta-analysis to summarize the current evidence.

**Methods:** We performed systematic review and meta-analysis of observational studies from January 1990 to March 2020 about the relation between BBB disruption and HT in patients with acute ischemic stroke with both computed tomography (CT) and magnetic resonance (MR) assessment of BBB. The outcome of interest was HT at follow-up imaging evaluation (within 48 h from symptom onset). We pooled data from available univariate odds ratios (ORs) in random-effects models with DerSimonian–Laird weights and extracted cumulative ORs.

**Results:** We included 30 eligible studies (14 with CT and 16 with MR), *N* = 2,609 patients, with 88% and 70% of patients included in CT and MR studies treated with acute stroke therapy, respectively. The majority of studies were retrospective and had high or unclear risk of bias. BBB disruption was measured with consistent methodology in CT studies, whereas in MR studies, there was more variability. All CT studies provided a BBB disruption cutoff predictive of HT. Four CT and 10 MR studies were included in the quantitative analysis. We found that BBB disruption was associated with HT with both CT (OR = 3.42; 95%CI = 1.62–7.23) and MR (OR = 9.34; 95%CI = 3.16–27.59). There was a likely publication bias particularly for MR studies.

**Conclusion:** Our results confirm that BBB disruption is associated with HT in both CT and MR studies. Compared with MR, CT has been more uniformly applied in the literature and has resulted in more consistent results. However, more efforts are needed for harmonization of protocols and methodology for implementation of BBB disruption as a neuroradiological marker in clinical practice.

## Introduction

Ischemic stroke is a major cause of death and disability all over the world. In the last decades, acute treatments aiming to recanalize the occluded vessel demonstrated efficacy in reducing the functional burden of the disease; however, reperfusion of the ischemic tissue brings some risk, with the most feared being hemorrhagic transformation (HT). HT is a common phenomenon after brain ischemia, occurs in up to 40% of patients treated with acute stroke therapy (1), and is fatal in around 3% of patients (2). Identification of factors predictive of HT is therefore important to stratify the hemorrhagic risk of patients and for management of the hyperacute stroke phase.

*In vitro* and *in vivo* models suggested failure of endothelial integrity and loss of neurovascular homeostasis as the cellular mechanisms underlying blood extravasation (3, 4) and, from a structural point of view, disruption of the blood–brain barrier (BBB) as the pathophysiological step that leads to HT (5, 6). *In vivo* visualization and measurement of BBB disruption in the acute stroke setting before reperfusion therapy may represent a useful marker to identify patients more prone to develop HT. BBB disruption can be evaluated with either computed tomography (CT) or magnetic resonance (MR). Within each imaging technique, BBB disruption can be investigated using different algorithms that mainly measure contrast extravasation through microcirculation, with either qualitative or quantitative methods (7).

Although diverse studies have provided data about the link between BBB disruption and HT, no conclusive evidence is available, and BBB disruption, although potentially a useful biomarker, has not yet been adopted in clinical practice with regard to acute ischemic stroke. To investigate the effect of BBB disruption on HT and provide more precise estimate of this effect, we performed a systematic review of studies that evaluated BBB disruption with either CT or MR in acute ischemic stroke setting and subsequent HT.

## Methods

This review was performed according to Preferred Reporting Items for Systematic Reviews and Meta-Analyses (PRISMA) (8) and Meta-analysis Of Observational Studies in Epidemiology (MOOSE) (9) recommendations and the Cochrane Handbook for Systematic Review of Interventions (https://training.cochrane.org/handbook). Data search, extraction, analysis, and interpretation were performed following a pre-specified study protocol developed by the investigators (not registered or published).

### Search Strategy and Selection Criteria

Potentially eligible studies were identified using PubMed and EMBASE databases by two independent investigators (FA and CR). Discrepancies were solved by consensus of all authors. We searched for eligible published studies in English, from January 1990 to March 2020, using the following search strategy: (“acute ischemic stroke” OR ((stroke OR “Acute Cerebrovascular Accident” OR “Acute Cerebrovascular Accidents”) AND (ischaemic OR ischemic))) AND ((hemorrhag^*^ OR haemorrhag^*^ OR “parenchymal hematoma” OR “Cerebral Hemorrhage”[Mesh]) AND (Transform^*^ OR Petechia^*^ OR subsequent OR infarct^*^)) AND (“computed tomography perfusion” OR “CT perfusion” OR “perfusion computed tomography” OR “perfusion CT” OR “computed tomographic perfusion” OR ((“Perfusion”[Mesh] OR “Perfusion Imaging”[MeSH Terms])AND “Tomography, X-Ray Computed”[Mesh])) for CT studies in PubMed; (“acute ischemic stroke” OR ((stroke OR “Acute Cerebrovascular Accident” OR “Acute Cerebrovascular Accidents”) AND (ischaemic OR ischemic))) AND ((hemorrhag^*^ OR haemorrhag^*^ OR “parenchymal hematoma” OR “Cerebral Hemorrhage”[Mesh]) AND (Transform^*^ OR Petechia^*^ OR subsequent OR infarct^*^)) AND ((“Magnetic Resonance Imaging”[Mesh] OR “NMR Imaging” OR “MR Tomography” OR “NMR Tomography” OR “Steady State Free Precession MRI” OR Zeugmatography OR “Proton Spin Tomography” OR “MRI Scans” OR “MRI Scan” OR “Spin Echo Imaging” OR “magnetization transfer” OR “magnetic resonance imaging” OR “magnetic resonance tomography” OR “mr imaging” OR MRI OR “magnetic resonance”) AND (permeability OR “blood-brain-barrier” OR BBB)).

The reference list of eligible studies was screened to identify additional publications suitable for our purposes not included in the original list. We applied the following inclusion criteria: (1) English-written articles; (2) patients with acute ischemic stroke; (3) studies with observational (retrospective or prospective) design; (4) patients treated or not with acute stroke therapy (i.e., intravenous thrombolysis, intra-arterial procedures, or both); (5) assessment of BBB disruption with CT or MR scan before any acute stroke treatment; (6) assessment of HT at the follow-up CT or MR scan within 48 h from the first scan; and (7) studies with more than 10 patients. Case reports, conference abstracts, study protocols, and unpublished studies were not included. We also excluded experimental or animal studies. Where studies had overlapping cohorts, only the study with the largest sample size was included. We included studies that evaluated BBB disruption as either continuous or categorical (dichotomized) variable. Assessment of BBB disruption was defined either as quantitative when a numerical value within a continuous scale was provided or as qualitative when a visual rating (e.g., presence vs. absence of contrast parenchymal enhancement) was provided. Localization of BBB disruption was defined as follows: “focal” when the BBB disruption was detected and measured only in a restricted area of the whole ischemic tissue and “global” when the BBB disruption was detected and measured in the whole ischemic tissue.

### Risk of Bias Assessment

Three investigators (CR, DC, and FV) independently extracted data from relevant studies using a predefined form including the following sections: (1) year of publication and study period; (2) study design; (3) inclusion and exclusion criteria; (4) clinical and radiological data; (5) definition and measurement of BBB; and (6) definition of HT. The same three investigators assessed study quality and risk of bias using the Newcastle-Ottawa Scale for cohort studies and the Cochrane “Tool to Assess Risk of Bias in Cohort Studies” (https://methods.cochrane.org/). In case of uncertainty, the final decision was taken by an expert (FA).

### Outcome

Our main outcome of interest was HT evaluated with CT or MR scan within 48 h from the first scan. We included studies with the following definitions of HT: (1) presence/absence; (2) ECASS-2 (European Co-operative Acute Stroke Study-II) (3) (10) NINDS (National Institute of Neurological Disorders and Stroke) (11) criteria; and (4) SITS-MOST (Safe Implementation of Thrombolysis in Stroke: Monitoring Study) (12) criteria.

### Statistical Analysis

Data were pooled in the meta-analysis when at least two studies had available data on the main outcome of interest, i.e., HT. In all analyses, we used a random-effects model with DerSimonian–Laird weights. The direction and strength of the association between BBB permeability and HT were quantified using crude (i.e., unadjusted) odds ratio (OR) and their corresponding 95% confidence intervals (CIs), with the inverse variance method for weighting. We therefore included in the quantitative analysis (i.e., meta-analysis) only studies with available unadjusted OR. Statistical heterogeneity was assessed with I^2^ statistics and visual inspection of forest plots. Values of ≤ 25, 25 to 50, and ≥50% were defined as low, moderate, and high degrees of heterogeneity, respectively. Publication bias was explored on funnel plots. All the analyses were performed in May 2020 using the meta-analysis software RevMan 5 (https://community.cochrane.org/).

### Data Availability

Requests to access the dataset from qualified researchers trained in human subject confidentiality protocols may be sent to the corresponding author.

## Results

**Figure 1:**
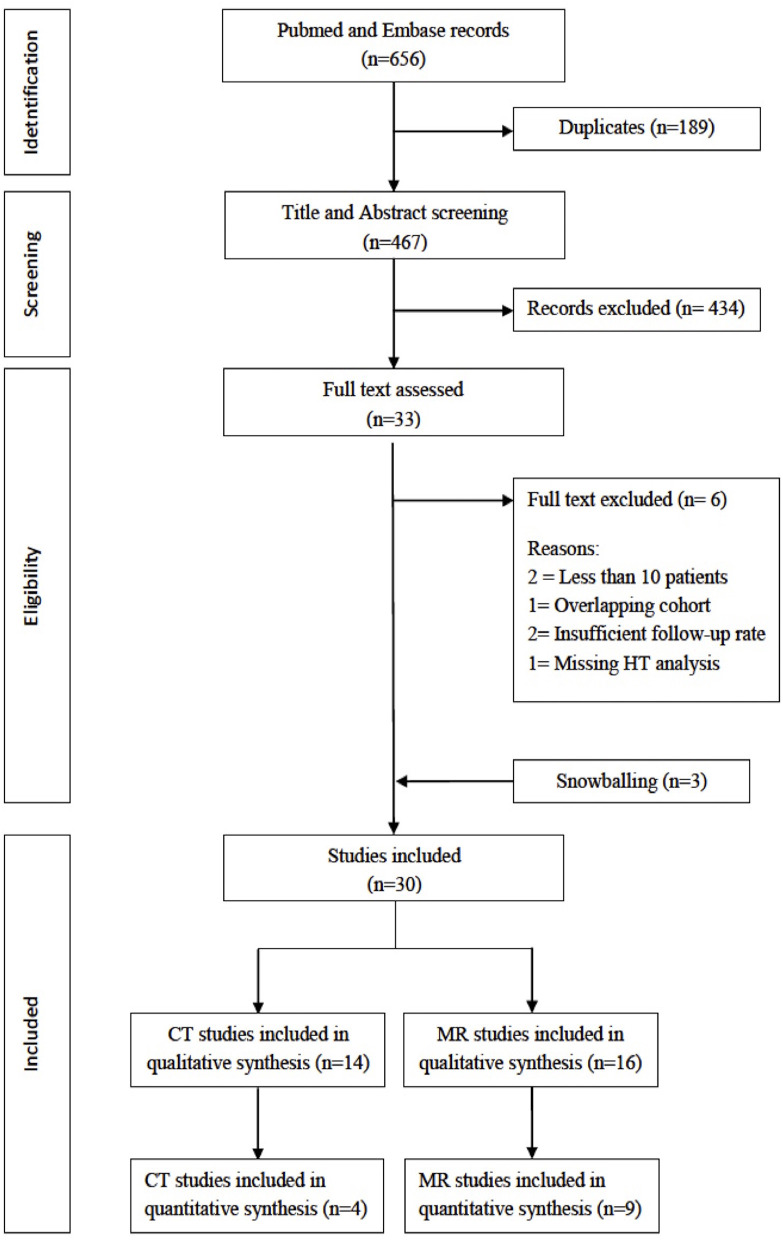
Preferred reporting items for systematic reviews and meta-analyses (PRISMA) flowchart of study selection.

The initial search retrieved 656 results. After removing 189 duplicates, we screened 467 titles and abstracts and excluded 434 articles. We therefore examined 33 full-text articles and excluded two studies for sample size <10 patients, one study for overlapping cohort, two studies for an inadequate follow-up rate, and one study for missing HT assessment. We retrieved three studies from reference snowballing; we therefore included in the systematic review 30 articles. Of these, 14 had BBB assessment with CT (13–26) and 16 with MR (27–42), with a total of 2,609 patients (1,510 with CT and 1,099 with MR). The study selection process is illustrated in Figure 1. Data for the quantitative analysis (meta-analysis) were available for 1,298 (50%) patients, from 4/14 studies with CT (794 patients, 53% of patients included in CT studies) and 9/16 MR studies (504 patients, 45% of patients included in MR studies). Clinical data of studies included in the meta-analysis are shown in Table 1.

**Table 1:** Summary of clinical data of studies included in meta-analysis.

**References**	**Design**	**Country and inclusion period**	**Sample size, *N***	**Acute treatment, *N***	**Stroke location, time from onset**	**Imaging type**	**BBB measurement type**	**BBB localization**	**Age, mean (±SD)**	**NIHSS, median (IQR) or mean (±SD)**	**HT, *N* (type)**
Arba et al. (26)	Prospective, SC	Italy, 2015–2018	171	32 i.v. rt-PA, 102 MT, 37 both	AC, <12 h	TC	Ktrans	F, G	76 (±12)	18 (12–23)	31 (HI-2, PH-1, PH-2), 12 sICH
Hom et al. (15)	Retrospective, SC	USA, 2006–2009	32	NA	AC, <12 h	TC	PS	G	72 (65–85)	14 (10–17)	3 (PH-2 = 3)
Horsch et al. (23)	Retrospective, MC	Netherlands, 2009–2013	545	501 i.v. rt-PA, 44 IAT and/or MT	NA, <9 h	TC	PS	NA	68 (58–77)	8 (4–13)	57 (HI-1 = 12, HI-2 = 17, PH-1 = 15, PH-2 = 13)
Kim et al. (24)	Prospective, SC	South Korea, 2013–2015	46	22 i.v. rt-PA, 12 MT, 12 both	AC, <6 h	TC	Ktrans	NA	66 (±12)	11 (8–16)	15 (HI-1 = 6, HI-2 = 2, PH-1 = 3, PH-2 = 4)
Bang et al. (29)	Retrospective, SC	USA, 2004–2006	32	13 i.v. rt-PA, 1 i.v. rt-PA+ IAT, 12 MT, 6 i.v. rt-PA+MT	MCA territory, NA	MRI	Gd enhancement	F	67 (±20)	NA	12 (HI-1 = 1, HI-2 = 2, PH-1 = 1, PH-2 = 5, SAH = 1, remote ICH = 1)
Hjort et al. (30)	Prospective, SC	Denmark, 2004–2006	33	33 i.v. rt-PA	MCA territory, <3 h	MRI	Gd enhancement	F	68 (±8)	11 (±6)	16 (HI = 13, PH = 3)
Kastrup et al. (32)	Retrospective, SC	Germany, NA	100	100 i.v. rt-PA	NA, (treated before <6 h)	MRI	Gd enhancement	F	67 (±14)	11 (7.5–15.5)	9 (PH-1 = 5, PH-2 = 4)
Kim et al. (28)	Retrospective, SC	Korea, 1997–2003	55	15 i.v. rt-PA, 40 no treatment	MCA territory, <6 h	MRI	Gd enhancement	G	68.8 (±10.8)	15.0 (±5.6)	19 (HI = 14, PH = 5)
Latour et al. (27)	Retrospective, SC	USA, 2000–2002	119	28 i.v. rt-PA, 1 i.a. rt-PA, 90 no treatment	NA, <24 h	MRI	Gd enhancement	F	72.3 (±13.5)	7.85 (±8.62)	22 (NA)
Lee et al. (35)	Retrospective, SC	USA, 2001–2009	14	1 i.v. rt-PA, 1 IAT, 8 MT, 4 both	PC, NA	MRI	Gd enhancement	F	71.1 (NA)	20.5 (range 0–36)	5 (HI-1 = 1, HI-2 = 2, PH2 = 1, intra-ventricular = 1)
Liu et al. (36)	Retrospective, SC	China, 2000–2004	26	26 no treatment	AC, NA	MRI	Ktrans	F	56.10 (±17.48)	NA	10 (NA)
Nael et al. (42)	Retrospective, MC	USA, 2004–2012	83	13 i.v. rt-PA, 23 MT, 18 both, 29 no treatment	AC, <8 h	MRI	K2	G	66 (±15.2)	17 (13–21)	20 (PH = 20)
Rozanski et al. (34)	Retrospective, SC	Germany, 2008	47	10 i.v. rt-PA, 37 no treatment	NA, <24 h	MRI	Gd enhancement	F	83.9 (NA)	5 (range 0–20)	8 (HI-1 = 0, HI-2 = 4, PH-1 = 1, PH-2 = 1, sICH = 2)
Leigh et al. (39)	Retrospective, MC	USA, Austria 2008–2011	100	47 MT, 53 MT+ i.v. rt-PA	NA, <12 h	MRI	K2	F	65.6 (NA)	15.1 (±NA)	57 (HI = 33, PH = 24)

*BB, blood–brain barrier; SC, single center; MC, multicenter; AC, anterior circulation; PC, posterior circulation; MCA, middle cerebral artery; rt-PA, recombinant tissue-plasminogen activator; i.v., intravenous; i.a., intra-arterial; IAT, intra-arterial thrombolysis; MT, mechanical thrombectomy; Gd, gadolinium; IQR, interquartile range; F, focal; G, global; SD, standard deviation; NIHSS, National Institute of Health Stroke Scale; HT, hemorrhagic transformation; HI, hemorrhagic infarction; PH, parenchymal hematoma; SAH, subarachnoid hemorrhage; ICH, intracranial hemorrhage; sICH, symptomatic intracranial hemorrhage; Ktrans, volume transfer constant; PS, permeability surface area products; rR, relative recirculation; %Recovery, percentage recovery; PB, post-bolus area; MPB, mean post-bolus intensity; CS, contrast slope; FC, final contrast; K2, tissue-to-blood transfer constant*.

### Computed Tomography Studies

Five studies were found as having a low risk of bias (15, 21, 23, 24, 26), seven a high risk (13, 17–20, 22, 25), and two an unclear risk (14, 16) (Supplemental Table 1). The general clinical characteristics of the included studies evaluating BBB with CT are summarized in the Supplemental Table 2. Studies were performed between 2004 and 2020; were from Europe (5), Asia (5), and North America (4); and were mainly single-center clinical cohorts, and five were with prospective design. Four studies had a sample size larger than 100 patients. Among 1,510 patients, a total of 359 (24%) had HT of any grade. The great majority of included patients (1,328/1,510; 88%) were treated with acute stroke therapy, including intravenous thrombolysis (*N* = 921), mechanical thrombectomy (*N* = 145), both (*N* = 58), and intra-arterial procedures (other than mechanical thrombectomy, *N* = 204); 150 (10%) patients received no treatment. For 32 (2%) patients, treatment type was not available. BBB assessment was performed within 24 h from symptom onset in all included studies. BBB disruption was measured with quantitative models in all studies: five studies used Ktrans, eight studies permeability surface (PS) products, and one study both parameters. Seven studies assessed focal BBB disruption, two studies global BBB disruption, and three studies both; in two studies, the localization of BBB assessment was not available. The radiological characteristics of the included studies are shown in Supplemental Table 3. Vendor largely varied across and within studies, slice number of scan acquisition ranged from 40 to 320, coverage ranged from 2.4 to 9 cm (five studies did not provide coverage), images were obtained with single-phase acquisition protocols in 10 studies and with a two-phase acquisition protocol in three studies, and acquisition time ranged across studies from 40 to 1,092 s. All CT studies performed a quantitative BBB disruption evaluation, which was made with the Patlak model in seven studies, with the non-linear-regression model in two studies and the Johnson–Wilson model in two studies, whereas in two studies, the model was not stated. Eleven studies provided a cutoff of BBB disruption predictive of HT (Supplemental Table 3), which ranged from 0.33 to 7 ml/100 mg/min in studies with Ktrans and from 0.23 to 6 ml/100 mg/min in studies with PS.

**Figure 2:**
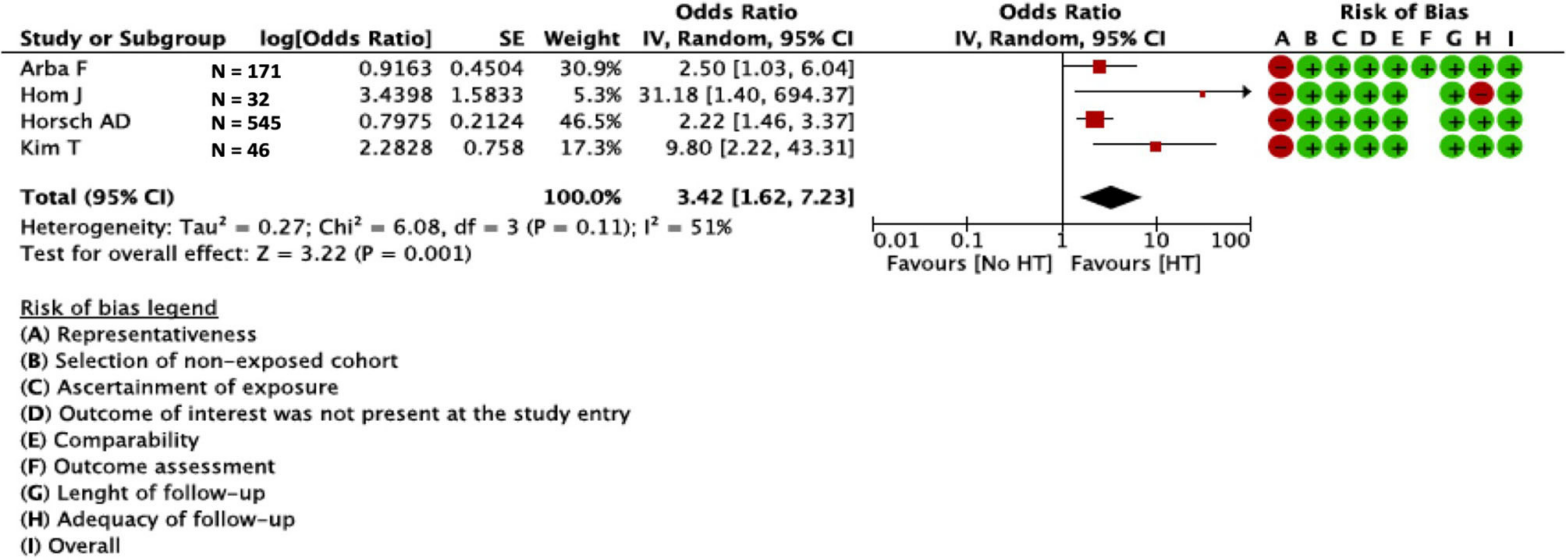
Relation between BBB and HT in CT studies. BBB, blood–brain barrier; HT, hemorrhagic transformation; CT, computed tomography.

Univariate ORs were present in four studies (*N* = 794)—two prospective (24, 26) and two retrospective (15, 23)—other studies provided only multivariate or did not provide ORs. In the meta-analysis of the four aforementioned studies, BBB disruption was associated with HT (OR = 3.42; 95%CI = 1.62–7.23). We found moderate statistical heterogeneity across studies (I^2^ = 51%; *p* = 0.001) (Figure 2). Visual inspection of funnel plots showed slight asymmetry, suggesting possible presence of publication bias Supplemental Figure 1; we did not perform further tests to explore publication bias since we pooled <10 studies.

### Magnetic Resonance Studies

Five studies were found as having a low risk of bias (27, 38, 39, 41, 42), eight a high risk (28–31, 33, 34, 37, 40), and three an unclear risk (32, 35, 36) (Supplemental Table 4). The general clinical characteristics of the included studies evaluating BBB with MR are summarized in the Supplemental Table 5. Studies were performed between 1997 and 2018 and were from North America (7), Europe (4), Asia (2), or international cohorts (2) and were mainly single-center clinical cohorts; all but one are with retrospective design. Three studies had a sample size larger than 100 patients. Among 1,099 patients, a total of 326 (29.7%) had HT of any grade. The majority of included patients (759/1099; 69%) were treated with acute stroke therapy, including intravenous thrombolysis (*N* = 502), mechanical thrombectomy (*N* = 146), both (*N* = 109), and intra-arterial procedures (not specified, *N* = 2); 340 (31%) patients received no acute treatment. In nine studies, BBB assessment was performed within 24 h from symptom onset, whereas in seven studies, information about time of BBB assessment was not available. Twelve studies assessed focal, and four studies global BBB disruption. The radiological characteristics of the included studies are shown in the Supplemental Table 6. Compared with CT studies, there was less variability in vendor type, but three studies did not state the vendor type. Imaging studies were performed mainly on 1.5-T scanners; in two studies, magnetic field was not available. Section thickness was 5 mm in the majority of studies, although it was not specified in five studies; acquisition time ranged from more than 60 s to nearly 5 min and was not stated in 10 studies. Six studies measured BBB disruption with qualitative parameters such as parenchymal enhancement or extravasation of contrast, whereas seven studies provided quantitative measurements of BBB disruption. Of the latter studies, two studies used the Patlak model, and five studies used first-pass T2^*^ method. Only four studies, using different BBB measurements, provided a BBB disruption cutoff predictive of HT.

**Figure 3:**
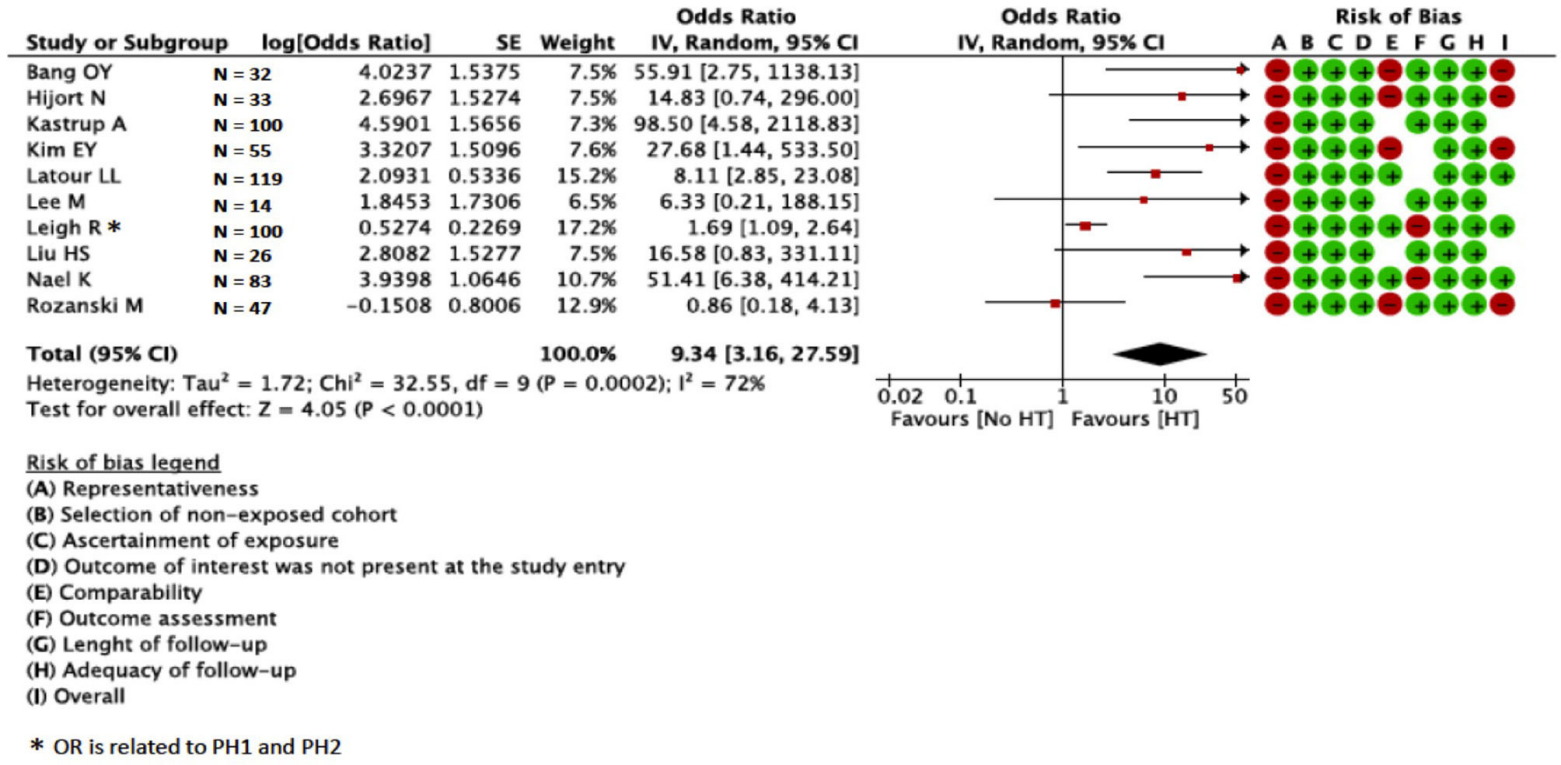
Relation between BBB and HT in MR studies. BBB, blood–brain barrier; HT, hemorrhagic transformation; MR, magnetic resonance.

Univariate ORs were present in 10 studies (*N* = 609)—one was prospective (30), nine were retrospective (27–29, 32–36, 39, 42), and three studies provided quantitative (*N* = 209) and seven qualitative (*N* = 400) BBB disruption assessment. Other studies provided only multivariate or did not provide OR. In the meta-analysis, BBB disruption was associated with HT (OR = 9.34; 95%CI = 3.16–27.59) (Figure 3). We found high statistical heterogeneity across studies (I^2^ = 72%; *p* < 0.00001). The association between BBB disruption and HT was confirmed in the sensitivity analysis for studies with qualitative assessment of BBB (OR = 9.96; 95%CI = 2.82–35.20; Supplemental Figure 2), whereas the association was not confirmed for studies with quantitative assessment (OR = 9.33; 95%CI = 0.76–114.39; Supplemental Figure 3). Visual inspection of funnel plots showed asymmetry suggesting presence of publication bias (Supplemental Figure 4); we did not perform further tests to explore publication bias since we pooled 10 studies.

## Discussion

In this systematic review, we brought together available observational studies regarding the relation between BBB disruption and HT in acute ischemic stroke patients. Overall, our results confirmed the association between BBB disruption and HT. While CT studies showed a three-fold increased risk of HT, MR studies showed a nine-fold increased risk of HT, with around six-fold increased difference in MR studies. Among studies included in the meta-analysis, only three (36, 39, 42) out of 10 MR studies had quantitative assessment of BBB disruption; MR studies had smaller sample size compared with CT studies, as reflected by the wider confidence intervals in the pooled MR analysis. Furthermore, in the sensitivity analysis for MR, quantitative BBB disruption was not associated with HT, whereas the association was confirmed in qualitative studies. It is important to note that the majority of the included studies were retrospective, with a high risk of bias and a likely publication bias; thus, our conclusions have limitations.

We found more MR studies with assessment of BBB, and the rate of HT was higher in MR studies, perhaps alluding to the higher sensitivity of MR in diagnosis of HT. By contrast, the pool of patients included in studies with CT was larger, and methodology and protocols were generally more consistent with each other. Patients enrolled in CT studies were more frequently treated with acute stroke therapy than those enrolled in MR studies. Given that acute stroke treatment increases the odds of HT occurrence, the results from CT studies mainly apply to patients treated with acute stroke therapy. Conversely, results from MR studies, although more generalizable to all ischemic stroke patients, included more heterogeneity with regard to treatment, with around a third of patients not treated with acute stroke treatment. While all CT studies provided a time frame for the study inclusion, around a half of MR had missing information about the time from onset of stroke to enrollment in the study. This is an important limitation, because extent of BBB disruption is thought to be time-dependent (43–45), and the timing of BBB assessment is therefore a pivotal information. Finally, occlusion site is fundamental for stroke therapy, since endovascular therapy proved efficacy only in anterior circulation (46), whereas in the posterior circulation, there is no conclusive evidence (47). Evaluation of BBB disruption may be challenging with CT in the posterior circulation due to the limits of perfusion technique in this area (48), whereas it is feasible with MR with dynamic contrast-enhanced sequences. However, the majority of CT studies enrolled patients with ischemic stroke in the anterior circulation, whereas the site of occlusion was not stated in many MR studies. As confirmation of the methodological variability of MR studies, we found a high statistical heterogeneity for MR studies compared with moderate heterogeneity for CT studies. Although we acknowledge that the precision of MR in detecting BBB disruption and HT is likely superior to CT, all those methodological pitfalls of MR studies limit considerably direct transferability of results into clinical practice.

There are diverse methods for evaluation of BBB disruption. BBB was measured with Ktrans or PS in CT studies, whereas MR studies adopted qualitative and quantitative methods, although some study did not specify how BBB was measured. Remarkably, all CT studies provided quantitative measurements with continuous values for BBB disruption, whereas around a half of MR studies provided qualitative evaluation of BBB, i.e., presence/absence of contrast leakage. BBB disruption is a dynamic process that varies with age, vascular risk factors such as diabetes and hypertension, and pre-existing characteristics of the brain (49, 50). Some degree of BBB disruption may be present up to 95% of patients with acute stroke within the ischemic area (51); thus, quantitative measurement of BBB disruption is useful to provide a precise estimate of HT risk. In order to differentiate pathologic from physiologic BBB leakage, it is also important to identify a cutoff value predictive of HT, since qualitative assessment of BBB disruption (i.e., presence/absence), although easily detectable, may not provide enough information to accurately estimate risk of subsequent HT. However, we observed a large inconsistency among cutoffs provided among diverse studies. Two meta-analyses were attempted to provide diagnostic accuracy of CT parameters, including BBB disruption, in predicting HT (52, 53). Although both studies found that BBB disruption has good sensitivity and specificity for HT prediction, there was high heterogeneity across studies due to several reasons, particularly differences in protocols and methods of CT perfusion for BBB evaluation. This is in keeping with our results that confirmed moderate and high statistical heterogeneity for CT and MR studies, respectively, and showed relevant differences across studies in qualitative analysis, highlighting the need of standardized and replicable protocols for BBB assessment in clinical setting.

Evaluation of BBB disruption may help in early stratification of hemorrhagic risk in patients with ischemic stroke, particularly those treated with intravenous thrombolysis and/or endovascular procedures (5, 40). HT extent may range from single-blood petechiae with few clinical consequences to symptomatic HT with a high rate of mortality and disability. While most studies reported the association between BBB disruption and HT, only few studies investigated the relation with unfavorable outcomes such as symptomatic intracranial hemorrhage (sICH) (20, 26, 34); therefore, this association, although potentially useful for clinicians, is still unclear and needs to be further clarified.

Our study has limitations that need to be addressed. Our quantitative analysis is based on unadjusted pooled estimates, and therefore, the association was not adjusted for other covariates and predictors of HT, such as age, stroke severity, and time from symptom onset to imaging. However, we extracted from the studies mean age of patients, stroke severity, site of occlusion, and time from onset to enrolment, and we observed that particularly in CT studies, such variables were similar, whereas in MR studies, there was more difference. It should be noted that only 4/14 CT studies (15, 23, 24, 26) and 10/16 MR studies (27–30, 32, 34–36, 42) reported univariate associations between BBB disruption and HT and were included in the meta-analysis; consequently, the ORs were found to represent a gross estimate of the relation between BBB and HT and should be interpreted with caution. Many CT and MR studies reported only adjusted associations; however, the sets of covariates largely differed across studies; thus, pooling-adjusted ORs were potentially inappropriate. This was also reflected by the number of studies with high or unclear risk of bias, mainly due to the adjusted analysis that often included covariates not relevant for the outcome of interest. Furthermore, funnel plots of studies included in the quantitative analysis suggested the presence of publication bias particularly for MR studies, possibly inflating the magnitude of effect of the association between BBB disruption and HT. Again, we examined studies from 1990 to 2020, and this may be a limitation due to the evolution in methods and technology for imaging in such a large time span. However, with a 30-year period of evaluation, our qualitative analysis is a comprehensive synthesis of available studies relevant for the topic, and the quantitative analysis attempted to provide ORs useful for future research. As a further limitation, we included only English-written studies.

More data are needed to overcome the limitations of current evidence about BBB disruption and HT. Our results suggest that protocols for BBB assessment need to increase consistency, BBB disruption evaluation should be quantitative, and future studies should provide a cutoff predictive of HT, preferably sICH or relevant HT. Furthermore, methodology and workflow of the studies should be easily reproducible and accessible. In this regard, as previously suggested (54, 55), CT seems to represent a fair trade-off between diagnostic detail and feasibility in acute stroke setting due to availability and few contraindications; however, MR is likely more accurate in detection of both BBB disruption and HT. Use of machine learning algorithms for MR (56) may help standardization of acquisition protocols and assessment of BBB in acute stroke; however, there is still no available evidence in this regard. Our results from the meta-analysis show that the association between BBB and HT is confirmed with both CT and MR, although with relevant limitations. Results from the systematic review suggest ease of standardized acquisition protocols, similar methodology, and similar characteristics of study population, which are the strengths of CT studies over MR, whereas the lack of standardized measurements for BBB disruption and quantitative cutoffs predictive of HT are the pitfalls of both CT studies and MR studies. Future studies need to define feasibility of protocols for BBB assessment and whether BBB disruption may serve as an adjunctive marker to identify patients at risk of HT, thus helping decision making and management of acute ischemic stroke patients.

## Data Availability Statement

The raw data supporting the conclusions of this article will be made available by the authors, without undue reservation.

## Author Contributions

FA conceived the study, contributed to the critical analysis of data, performed the statistical analysis, and drafted the paper. CR, DC, and FV contributed to the critical analysis of data, selected the studies, and drafted the paper. GB and EF contributed to the critical analysis of data and drafted the paper. All authors reviewed and approved the manuscript.

## Conflict of Interest

The authors declare that the research was conducted in the absence of any commercial or financial relationships that could be construed as a potential conflict of interest.
