# Supplementary material for: Blood–Brain Barrier Disruption and Hemorrhagic Transformation in Acute Ischemic Stroke: Systematic Review and Meta-Analysis
Source: Front Neurol. 2021 Jan 21;11:594613. doi: 10.3389/fneur.2020.594613 (PMC7859439; doi:10.3389/fneur.2020.594613)
Supplement: Supplementary file 1 [file Table_1.docx]

**Supplemental Table 1.** Risk of bias assessment of CT studies.

| **Study** | **1** | **2** | **3** | **4** | **5** | **6** | **7** | **8** | **Overall** |
| --- | --- | --- | --- | --- | --- | --- | --- | --- | --- |
| **Lin et al. (13)** | High | Low | Low | Low | High | High | Low | Low | High |
| **Aviv et al. (14)** | Unclear | Low | Low | Low | Unclear | Low | Low | Low | Unclear |
| **Hom et al. (15)** | High | Low | Low | Low | Low | High | Low | Low | Low |
| **Ozkul-wermester et al. (16)** | High | low | Low | Low | Unclear | Low | Low | Low | Unclear |
| **Bennink et al. (17)** | High | Low | Low | Low | High | high | Low | Low | High |
| **Yen et al. (18)** | Unclear | Low | Low | Low | High | High | Low | Low | High |
| **Chen et al. (19)** | High | Low | Low | Low | High | High | Low | Low | High |
| **Li et al. (20)** | High | Low | Low | Low | High | Low | Low | Low | High |
| **Li et al. (21)** | High | Low | Low | Low | Low | Low | Low | Low | Low |
| **Puig et al. (22)** | High | Low | Low | Low | High | Low | Low | Low | High |
| **Horsch et al. (23)** | High | Low | Low | Low | Low | Low | Low | Low | Low |
| **Kim et al. (24)** | High | Low | Low | Low | Low | Low | Low | Low | Low |
| **Li et al. (25)** | Unclear | Low | Low | Low | High | Low | Low | Low | High |
| **Arba et al. (26)** | High | Low | Low | Low | Low | Low | Low | Low | Low |

Newcastle-Ottawa Scale for cohort studies. 1. Representativeness of the study cohort; 2. Selection of the non-exposed cohort; 3. Ascertainment of exposure; 4. Demonstration that outcome of interest was not present at the study entry; 5. Comparability (were prognostic factors controlled for?); 6. Assessment of outcome; 7. Length of follow-up; 8. Adequacy of follow-up.

**Figure.** Risk of Bias assessment for studies with blood brain barrier disruption evaluated with Computed Tomography.
