# Supplementary material for: Blood–Brain Barrier Disruption and Hemorrhagic Transformation in Acute Ischemic Stroke: Systematic Review and Meta-Analysis
Source: Front Neurol. 2021 Jan 21;11:594613. doi: 10.3389/fneur.2020.594613 (PMC7859439; doi:10.3389/fneur.2020.594613)
Supplement: Supplementary file 2 [file Table_2.docx]

**Supplemental Table 2.** Summary of clinical data of studies with BBB assessment with CT perfusion.

| **Author** | **Design** | **Country and**  **Inclusion Period** | **Sample Size, N** | **Acute treatment** | **Stroke localization, time from onset** | **BBB measurement type** | **BBB localization** | **Age,**  **Median (IQR) or Mean (±SD)** | **NIHSS,**  **Median (IQR) or Mean (±SD)** | **HT,**  **N (type)** |
| --- | --- | --- | --- | --- | --- | --- | --- | --- | --- | --- |
| Lin et al. (13) | Retrospective, SC | USA,  2004-2006 | 50 | 16 i.v. rt-PA, 2 i.a rt-PA, 32 no treatment | AC, PC, <3 h | PS | F | NA | NA | 6 (HI-1=1, HI-2=2, PH-1=3) |
| Aviv et al. (14) | Prospective, SC | Canada,  2006-2007 | 41 | 20 i.v. rt-PA, 2 i.a. rt-PA, 19 no treatment | NA, <3 h | PS | F | 70 (±15) | 15 (7-20) | 23 (HI=15, PH=8) |
| Hom et al. (15) | Retrospective, SC | USA,  2006-2009 | 32 | NA | AC, <12 h | PS | G | 72 (65-85) | 14 (10-17) | 3 (PH-2=3) |
| Ozkul-Wermester et al. (16) | Prospective, SC | France,  2009-2010 | 86 | 31 i.v. rt-PA, 55 no treatment | NA, <6 h | PS | F | NA | NA | 27 (HI-1=6, HI-2=10, PH-1=8, PH-2=3) |
| Bennink et al. (17) | Retrospective, MC | Netherlands,  2009-2013 | 60 | 55 i.v. rt-PA, 4 i.a. rt-PA or MT, 1 no treatment | AC, <9 h | Ktrans, PS | F, G | 69 (±13) | 11±6 | 20 (NA) |
| Yen et al. (18) | Retrospective, SC | Canada,  NA | 42 | 33 i.v. rt-PA, 5 i.v. rt-PA+MT, 4 no treatment | AC, <6 h | PS | F | 70 (±13) | NA | 15 (HI=8, PH/SAH=7) |
| Chen et al. (19) | Retrospective, MC | China,  2011-2014 | 41 | 41 IAT | AC, <9 h | Ktrans | F | 64 (±13) | 16±4 | 41 (HI=26, PH=15) |
| Li et al. (20) | Retrospective, MC | China,  2011-2014 | 106 | 106 IAT | AC, <9 h | Ktrans | F, G | 65 (±14) | 16 (12-19) | 48 (sICH=21) |
| Li et al. (21) | Retrospective, MC | China,  2011-2016 | 70 | 40 i.v. rt-PA, 11 MT, 9 both, 10 no treatment | AC, <6 h | PS | F | NA | NA | 14 (PH-1=2, PH-2=12) |
| Puig et al. (22) | Prospective, SC | Spain,  2012-2016 | 156 | 156 i.v. rt-PA | AC, <4.5 h | Ktrans | F | 75 (66-81) | 13 (7-19) | 37 (HI-1=10, HI-2=7, PH-1=8, PH-2=12) |
| Horsch et al. (23) | Retrospective, MC | Netherlands,  2009-2013 | 545 | 501 i.v rt-PA, 44 IAT and/or MT | NA, <9 h | PS | NA | 68 (58-77) | 8 (4-13) | 57 (HI-1=12, HI-2= 17, PH-1=15, PH-2=13) |
| Kim et al. (24) | Prospective, SC | South Korea,  2013-2015 | 46 | 22 i.v. rt-PA, 12 MT, 12 both | AC, <6 h | Ktrans | NA | 66 (±12) | 11 (8-16) | 15 (HI-1= 6, HI-2=2, PH-1=3, PH-2=4) |
| Li et al. (25) | Retrospective, SC | China,  2017-2019 | 64 | 15 i.v. rt-PA, 20 MT, 29 no treatment | AC, <24 h | PS | G | 65 (±14) | NA | 22 (NA) |
| Arba et al. (26) | Prospective, SC | Italy,  2015-2018 | 171 | 32 i.v. rt-PA, 102 MT, 37 both | AC, <12 h | Ktrans | F, G | 76 (±12) | 18 (12-23) | 31 (HI-2, PH-1, PH-2), 12 sICH |

BBB=Blood-Brain Barrier; SC=Single Center; MC=Multi Center; AC=Anterior Circulation; PC=Posterior Circulation; PS=Permeability Surface area products; Ktrans = transfer Constant; NA=Not Available; rt-PA=recombinant tissue-Plasminogen Activator; MT=Mechanical Thrombectomy; i.v.=intravenous; i.a.= intraarterial; IAT= Intra-Arterial Thrombolysis; F=Focal; G=Global; IQR= Interquartile Range; SD=Standard Deviation; NIHSS= National Institute of Health Stroke Scale; HT=Hemorrhagic Transformation; HI=Hemorrhagic Infarction; PH=Parenchymal Hematoma; SAH: Subarachnoid Hemorrhage; sICH= symptomatic Intracranial Hemorrhage
