# Supplementary material for: Blood–Brain Barrier Disruption and Hemorrhagic Transformation in Acute Ischemic Stroke: Systematic Review and Meta-Analysis
Source: Front Neurol. 2021 Jan 21;11:594613. doi: 10.3389/fneur.2020.594613 (PMC7859439; doi:10.3389/fneur.2020.594613)
Supplement: Supplementary file 3 [file Table_3.docx]

**Supplemental Table 3.** Summary of radiological data of studies with BBB assessment with CT perfusion.

| **Author** | **Vendor** | **Slice numbers** | **Acquisition phase/s** | **Section thickness (mm)** | **Acquisition time (sec)** | **Coverage (cm)** | **Injection rate (ml/sec)** | **Permeability model** | **BBB assessment** | **BBB parameters and cut-offs*** |
| --- | --- | --- | --- | --- | --- | --- | --- | --- | --- | --- |
| Lin et al. (13) | Siemens Healthcare | 16 | One | 12 | 60 | 2.4 | 4-5 | Patlak | Quantitative | PS > 5.16-5.99 |
| Aviv et al. (14) | GE Healthcare | 64 | Two | 5 | 135 | 4 | 4 | Johnson and Wilson | Quantitative | PS > 0.23 |
| Hom et al. (15) | Philips Healthcare | 64 | Two | 5 | 90 to 240 | 10 | 5 | Patlak | Quantitative | Ktrans > 5 |
| Ozkul-Wermester et al. (16) | GE Healthcare | 64 | Two | 1.25 | 150 | 4 | 4 | Johnson and Wilson | Quantitative | PS > 0.84 |
| Bennink et al. (17) | Philips Healthcare, Siemens Healthcare,  GE Healthcare, Toshiba Medical System | 40 to 256 | One | 5 | at least 210 | 4-6.5 | 6 | NLR, Patlak | Quantitative | rKtrans |
| Yen et al. (18) | Siemens Healthcare | 128 | One | 5 | 105 | 9.6 | 5 | Patlak | Quantitative | rPS > 1.3  PS |
| Chen et al. (19) | GE Healthcare | 64 | One | NA | NA | 2 | 5 | Patlak | Quantitative | Ktrans > 0.334 |
| Li et al. (20) | GE Healthcare | 64 | One | NA | 50-70 | 4 | 4 | Patlak | Quantitative | Ktrans > 0.35 |
| Li et al. (21) | Philips Healthcare, GE Healthcare | 256  64 | One | NA | NA | Whole-brain | 4.5 | Johnson and Wilson | Quantitative | PS > 1.179  rPS > 2.89 |
| Puig et al. (22) | Philips Healthcare | 128 | One | 10 | 220 | 4 | 5 | Patlak | Quantitative | Ktrans > 7 |
| Horsch et al. (23) | Philips Healthcare, Siemens Healthcare, GE Healthcare, Toshiba Medical System | 40 to 320 | One | 5 | 210 | NA | 6 | NLR | Quantitative | rPS |
| Kim et al. (24) | Toshiba Medical System | 64 | NA | NA | NA | NA | NA | NA | Quantitative | BBBP > 7 |
| Li et al. (25) | GE Healthcare | 256 | One | 5 | NA | NA | 6 | Johnson and Wilson | Quantitative | PS > 0.79 |
| Arba et al. (26) | Philips, Siemens | 128 | One | 5 | 50 | 8 | 5 | Patlak | Quantitative | Ktrans >0.63 |

*=ml/100mg/min unless otherwise specified

BBB=Blood-Brain Barrier; NA=Not Available; NLR=Non Linear Regression; PS = Permeability Surface area product; rPS = relative Permeability Surface area product (relative to contralateral hemisphere); Ktrans = transfer Constant; rKtrans = relative transfer Constant (relative to contralateral hemisphere); BBBP = BBB Permeability
