# Supplementary material for: Blood–Brain Barrier Disruption and Hemorrhagic Transformation in Acute Ischemic Stroke: Systematic Review and Meta-Analysis
Source: Front Neurol. 2021 Jan 21;11:594613. doi: 10.3389/fneur.2020.594613 (PMC7859439; doi:10.3389/fneur.2020.594613)
Supplement: Supplementary file 4 [file Table_4.docx]

**Supplemental Table 4.** Risk of bias assessment of MR studies.

| **Study** | **1** | **2** | **3** | **4** | **5** | **6** | **7** | **8** | **Overall** |
| --- | --- | --- | --- | --- | --- | --- | --- | --- | --- |
| **Latour et al. (27)** | High | Low | Low | Low | Low | Low | Low | Low | Low |
| **Kim et al. (28)** | High | Low | Low | Low | High | Low | Low | Low | High |
| **Bang et al. (29)** | High | Low | Low | Low | High | Low | Low | Low | High |
| **Hjort et al. (30)** | High | Low | Low | Low | High | Low | Low | Low | High |
| **Kassner et al. (31)** | High | Low | Low | Low | High | Low | Unclear | Low | High |
| **Kastrup et al. (32)** | High | Low | Low | Low | Unclear | Low | Low | Low | Unclear |
| **Thornhill et al. (33)** | High | Low | Low | Low | High | High | Low | Low | High |
| **Rozanski et al. (34)** | High | Low | Low | Low | High | Low | Low | Low | High |
| **Lee et al. (35)** | High | Low | Low | Low | Unclear | Low | Low | Low | Unclear |
| **Liu et al. (36)** | High | Low | Low | Low | Unclear | Low | Low | Low | Unclear |
| **Scalzo et al. (37)** | High | Low | Low | Low | High | Low | Low | Low | High |
| **Leigh et al. (38)** | High | Low | Low | Low | Low | High | Low | Low | Low |
| **Leigh et al. (39)** | High | Low | Low | Low | Low | High | Low | Low | Low |
| **Simpkins et al. (40)** | High | Low | Low | Low | High | Low | Low | Low | High |
| **Villringer et al. (41)** | High | Low | Low | Low | Low | Unclear | Low | Low | Low |
| **Nael et al (42)** | High | Low | Low | Low | Low | High | Low | Low | Low |

Newcastle-Ottawa Scale for cohort studies. 1. Representativeness of the study cohort; 2. Selection of the non-exposed cohort; 3. Ascertainment of exposure; 4. Demonstration that outcome of interest was not present at the study entry; 5. Comparability (were prognostic factors controlled for?); 6. Assessment of outcome; 7. Length of follow-up; 8. Adequacy of follow-up.
