# Supplementary material for: Blood–Brain Barrier Disruption and Hemorrhagic Transformation in Acute Ischemic Stroke: Systematic Review and Meta-Analysis
Source: Front Neurol. 2021 Jan 21;11:594613. doi: 10.3389/fneur.2020.594613 (PMC7859439; doi:10.3389/fneur.2020.594613)
Supplement: Supplementary file 5 [file Table_5.docx]

**Supplemental Table 5.** Summary of clinical data of studies with BBB assessment with MR.

| **Author** | **Design** | **Country and**  **inclusion period** | **Sample size,**  **N** | **Acute treatment,**  **N** | **Stroke location, time from onset** | **BBB measurement type** | **BBB localization** | **Age,**  **mean (±SD)** | **NIHSS, median (IQR) or mean (±SD)** | **HT,**  **N (type)** |
| --- | --- | --- | --- | --- | --- | --- | --- | --- | --- | --- |
| Latour et al. (27) | Retrospective, SC | USA,  2000-2002 | 119 | 28 i.v. rt-PA,  1 i.a. rt-PA, 90  no treatment | NA,  <24h | Gd enhancement | F | 72.3 (±13.5) | 7.85 (±8.62) | 22 (NA) |
| Kim et al. (28) | Retrospective, SC | Korea,  1997-2003 | 55 | 15 i.v. rt-PA, 40 no treatment | MCA territory,  <6h | Gd enhancement | G | 68.8 (±10.8) | 15.0 (±5.6) | 19 (HI=14, PH=5) |
| Bang et al. (29) | Retrospective, SC | USA,  2004-2006 | 32 | 13 i.v. rt-PA, 1 i.v. rt-PA+ IAT, 12 MT, 6 i.v rt-PA+MT | MCA territory,  NA | Gd enhancement | F | 67 (±20) | NA | 12 (HI-1=1, HI-2=2, PH-1=1, PH-2=5, SAH=1, remote ICH=1) |
| Hjort et al. (30) | Prospective, SC | Denmark,  2004-2006 | 33 | 33 i.v. rt-PA | MCA territory,  <3h | Gd enhancement | F | 68 (±8) | 11 (±6) | 16 (HI=13, PH=3) |
| Kassner et al. (31) | Retrospective, SC | Canada,  NA | 36 | 15 i.v. rt-PA, 21 no treatment | NA,  NA | KPS | G | 67.7 (±15.2) | 8.26 (±4.69) | 13 (HI-1=2, HI-2=3, PH-1=4, PH-2=4) |
| Kastrup et al. (32) | Retrospective, SC | Germany,  NA | 100 | 100 i.v. rt-PA | NA,  (treated before<6h) | Gd enhancement | F | 67 (±14) | 11 (7.5-15.5) | 9 (PH-1=5, PH-2=4) |
| Thornhill et al. (33) | Retrospective, SC | Canada,  NA | 18 | 8 i.v. rt-PA, 10 no treatment | NA,  <7h | rR, Peak Height, %Recovery, Slope (DSC-MRI), Ktrans (DCE-MRI) | F | 68 (±17.5) | NA | 8 (HI-1=3, HI-2=1; PH-1=2, PH-2=2) |
| Rozanski et al. (34) | Retrospective, SC | Germany,  2008 | 47 | 10 i.v. rt-PA, 37 no treatment | NA,  <24h | Gd enhancement | F | 83.9 (NA) | 5 (range 0-20) | 8 (HI-1=0, HI-2=4, PH-1=1, PH-2=1, sICH=2) |
| Lee et al. (35) | Retrospective, SC | USA,  2001-2009 | 14 | 1 i.v. rt-PA, 1 IAT,  8 MT, 4 both | PC,  NA | Gd enhancement | F | 71.1 (NA) | 20.5 (range 0-36) | 5 ( HI-1=1, HI-2=2, PH2=1, intra-ventricular= 1) |
| Liu et al. (36) | Retrospective, SC | China,  2000-2004 | 26 | 26 no treatment | AC,  NA | Ktrans | F | 56.10 (±17.48) | NA | 10 (NA) |
| Scalzo et al. (37) | Retrospective, MC | USA, Canada, UK,  NA | 263 | 129 i.v. rt-PA,  55 MT,  27 i.v. rt-PA+ MT,  52 no treatment | NA,  NA | rR, %Recovery, post-bolus area(PB) ,mean post-bolus  intensity(MPB), contrast slope(CS), final contrast (FC) | G | 69 (±15) | 10 (range 0-40) | 84 (HI1=34, HI-2=30, PH-1=9 PH-2=11) |
| Leigh et al.  (38) | Retrospective, MC | NA,  NA | 75 | 75 i.v. rt-PA | NA,  NA | K2 | F | 70 (±17) | 12 (±9) | 28 (HI=19, PH=9) |
| Leigh et al. (39) | Retrospective, MC | USA, Austria,  2008-2011 | 100 | 47 MT, 53 MT + i.v. rt-PA | NA,  <12h | K2 | F | 65.6 (NA) | 15.1 (±NA) | 57 (HI=33, PH=24) |
| Simpkins et al. (40) | Retrospective, SC | USA,  2013-2014 | 43 | 43 i.v. rt-PA | NA,  NA | K2 | F | 70 (NA) | 8 (±NA) | 4 (PH=4) |
| Villringer et al. (41) | Retrospective, SC | Germany,  2008-2013; 2014-2018 | 54 | 19 i.v. rt-PA,  35 no treatment | NA,  <24h | Ktrans | F | 70 (±12) | 4 (2–8) | 11 (HI1=6, HI2=5) |
| Nael et al. (42) | Retrospective, MC | USA,  2004-2012 | 83 | 13 i.v. rt-PA,  23 MT, 18  both, 29  no treatment | AC,  <8h | K2 | G | 66 (±15.2) | 17 (13–21) | 20 (PH=20) |

BBB=Blood-Brain Barrier; SC=Single Center; MC=Multi Center; AC=Anterior Circulation; PC=Posterior Circulation; MCA=Middle Cerebral Artery; NA= Not Available; rt-PA=recombinant tissue-Plasminogen Activator; i.v.=intravenous; i.a.=intraarterial; IAT= Intra-Arterial Thrombolysis; MT=Mechanical Thrombectomy; Gd= Gadolinium; IQR=Interquartile range; F=Focal; G=Global; SD= Standard Deviation; NIHSS= National Institute of Health Stroke Scale; HT=Hemorrhagic Transformation; HI=Hemorrhagic Infarction; PH=Parenchymal Hematoma; SAH= Subarachnoid Hemorrhage; ICH: Intracranial Hemorrhage; sICH: Symptomatic Intracranial Hemorrhage; Ktrans= Volume transfer constant; rR= relative recirculation; %Recovery= percentage recovery; PB= post-bolus area; MPB= mean post-bolus intensity; CS= contrast slope; FC= final contrast; K2= tissue-to-blood transfer constant.
