# Supplementary material for: Blood–Brain Barrier Disruption and Hemorrhagic Transformation in Acute Ischemic Stroke: Systematic Review and Meta-Analysis
Source: Front Neurol. 2021 Jan 21;11:594613. doi: 10.3389/fneur.2020.594613 (PMC7859439; doi:10.3389/fneur.2020.594613)
Supplement: Supplementary file 6 [file Table_6.docx]

**Supplemental Table 6.** Summary of radiological data of studies with BBB assessment with MR.

| **Author** | **Vendor** | **Magnetic field (T)** | **Section thickness (mm)** | **Acquisition time (sec)** | **Injection rate (ml/sec)** | **Permeability model** | **BBB acquisition sequences** | **BBB assessment** | **BBB parameters and cut-offs** |
| --- | --- | --- | --- | --- | --- | --- | --- | --- | --- |
| Latour et al. (27) | NA | 1.5 | NA | NA | NA | NA | FLAIR | Qualitative | PE |
| Kim et al. (28) | GE | 1.5 | 5 | NA | 4 | NA | T1 | Qualitative | PE |
| Bang et al. (29) | Siemens | 1.5 | 7 | NA | 5 | First pass T2* | DSC-T2 | Qualitative | NA |
| Hjort et al. (30) | GE | 1.5 or 3 | NA | NA | NA | NA | FLAIR | Qualitative | PE |
| Kassner et al. (31) | GE | 1.5 | 5 | 288 | 5 | Patlak | DCE-T1 | Quantitative | Ktrans = 0.67 ml/100g/min |
| Kastrup et al. (32) | Siemens | 1.5 | 5 | NA | NA | NA | T1 | Qualitative | PE |
| Thornhill et al. (33) | GE | 1.5 | 5 | 374 | 5 | First pass T2*  Patlak | DSC-T2, DCE-T1 | Quantitative  Quantitative | rR, %Recovery Ktrans |
| Rozanski et al. (34) | Siemens | 3 | 5 | NA | 5 | NA | FLAIR | Qualitative | PE |
| Lee et al. (35) | Siemens | 1.5 | 5-7 | >60 | 5 | First pass T2* | DSC-T2 | Qualitative | Increased signal intensity |
| Liu et al. (36) | Siemens | 1.5 | NA | NA | 4-5 | First pass T2* | DCE-T1 | Quantitative | Ktrans |
| Scalzo et al. (37) | Multibrand | 1.5 or 3 | 5 (4-7) | >60 | 5 | First pass T2* | DSC-T2 | Quantitative | rR, %Recovery, PB, MPB, CS, FC |
| Leigh et al.  (38) | GE  Philips | 1.5  3 | 7 | NA | NA | First pass T2* | DSC-T2 | Quantitative | K2, 21%* |
| Leigh et al. (39) | GE  Philips | 1.5  3 | 7 | NA | NA | First pass T2* | DSC-T2 | Quantitative | K2, 21%* |
| Simpkins et al. (40) | NA | NA | NA | NA | NA | First pass T2* | DSC-T2 | Quantitative | K2 |
| Villringer et al. (41) | Siemens | 3 | 5 | 118 | 1 | Patlak | DCE-T1 | Quantitative | Ktrans |
| Nael et al. (42) | NA | NA | NA | NA | NA | First pass T2* | DSC-T2 | Quantitative | K2= 0.28 |

BBB= Blood-Brain Barrier; NA= Not Available; PE= Parenchymal Enhancement; DSC= Dynamic Susceptibility Contrast; DCE= Dynamic Contrast Enhanced; Ktrans= Volume transfer constant; rR= relative recirculation; %Recovery= percentage recovery; Increased signal intensity= increased signal intensity at later time points in perfusion MRI acquisition, indicating local accumulation of contrast caused by BBB leakage; PB= post-bolus area; MPB= mean post-bolus intensity; CS= contrast slope; FC= final contrast; K2= tissue-to-blood transfer constant.

* 21% of mean Permeability Derangement
