# Supplementary figures and images for: Blood–Brain Barrier Disruption and Hemorrhagic Transformation in Acute Ischemic Stroke: Systematic Review and Meta-Analysis
Source: Front Neurol. 2021 Jan 21;11:594613. doi: 10.3389/fneur.2020.594613 (PMC7859439; doi:10.3389/fneur.2020.594613)

**Supplemental Figure 1.** Funnel plot of CT studies.

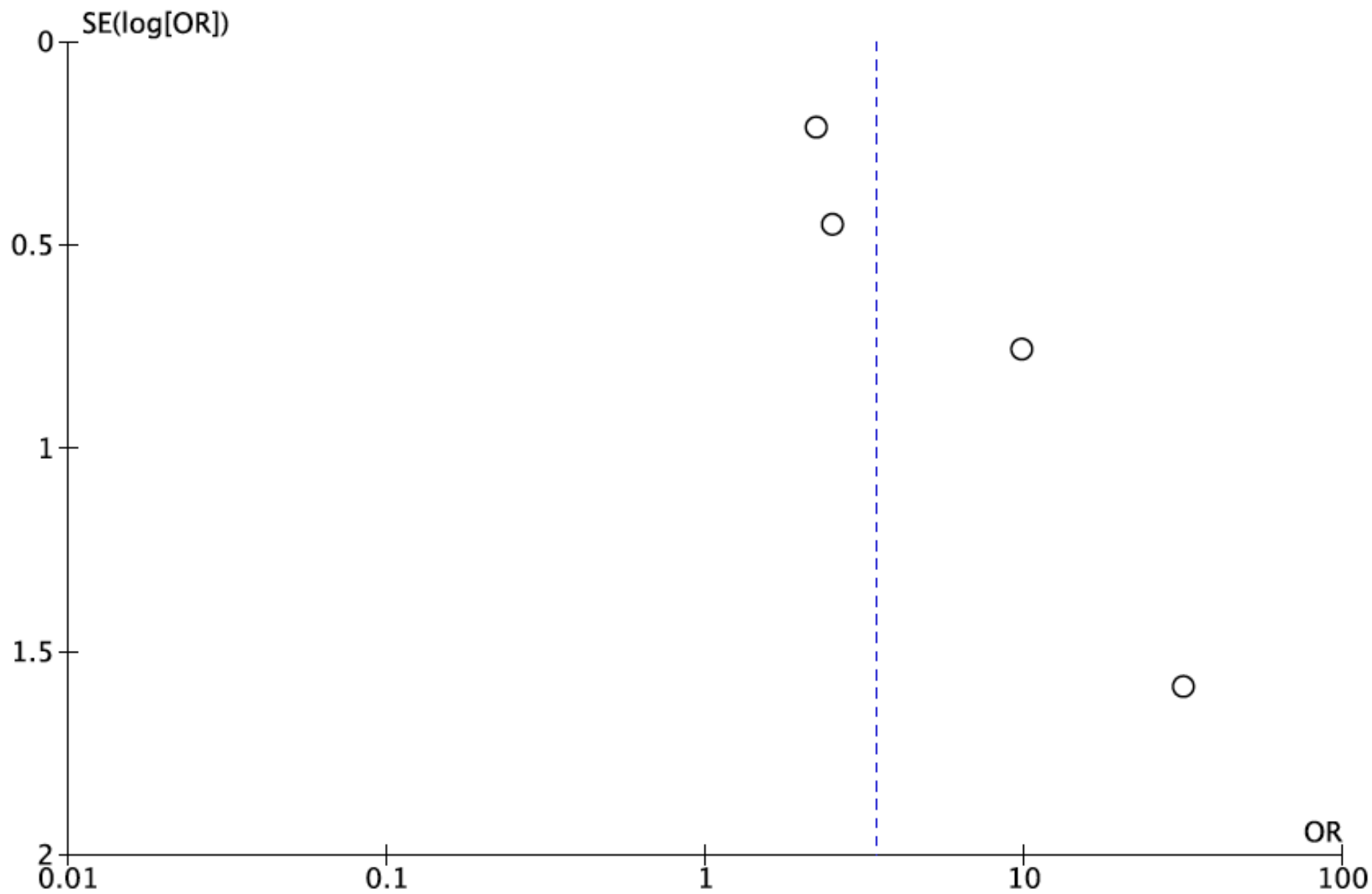

Supplement: Supplementary file 7 [file Image_1.pdf]

**Supplemental Figure 4.** Funnel plot of MR studies.

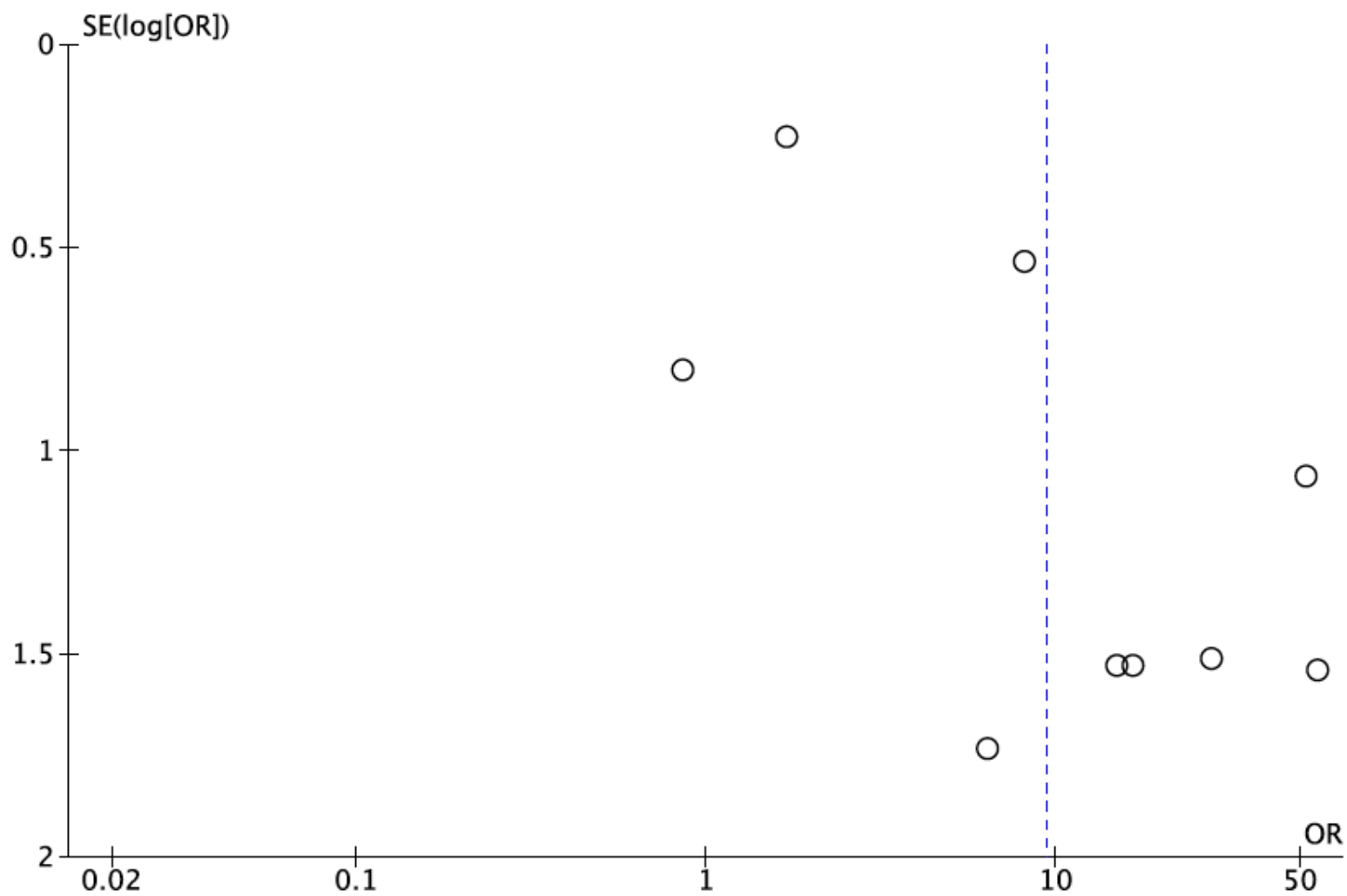

Supplement: Supplementary file 10 [file Image_4.pdf]
