# Supplementary material for: Blood–Brain Barrier Disruption and Hemorrhagic Transformation in Acute Ischemic Stroke: Systematic Review and Meta-Analysis
Source: Front Neurol. 2021 Jan 21;11:594613. doi: 10.3389/fneur.2020.594613 (PMC7859439; doi:10.3389/fneur.2020.594613)
Supplement: Supplementary file 8 [file Image_2.pdf]

Supplemental Figure 2. Relation between BBB (qualitative assessment) and HT in MR studies. BBB, blood–brain barrier; HT, hemorrhagic transformation; MR, magnetic resonance.

Study or Subgroup	log[Odds Ratio]	SE	Weight	Odds Ratio IV, Random, 95% CI	A	B	C	D	E	F	G	H	I
Bang OY	4.0237	1.5375	11.1%	55.91 [2.75, 1138.13]	−	+	+	+	−	+	+	+	−
Hijort N	2.6967	1.5274	11.2%	14.83 [0.74, 296.00]	−	+	+	+	−	+	+	+	−
Kastrup A	4.5901	1.5656	10.9%	98.50 [4.58, 2118.83]	−	+	+	+		+	+	+	
Kim EY	3.3207	1.5096	11.4%	27.68 [1.44, 533.50]	−	+	+	+	−		+	+	−
Latour LL	2.0931	0.5336	25.2%	8.11 [2.85, 23.08]	−	+	+	+	+		+	+	+
Lee M	1.8453	1.7306	9.5%	6.33 [0.21, 188.15]	−	+	+	+		+	+	+	
Rozanski M	−0.1508	0.8006	20.7%	0.86 [0.18, 4.13]	−	+	+	+	−	+	+	+	−
**Total (95% CI)**			**100.0%**	**9.96 [2.82, 35.20]**									

Heterogeneity: $Tau^2 = 1.36$; $Chi^2 = 12.69$, df = 6 (P = 0.05); $I^2 = 53\%$

Test for overall effect: Z = 3.57 (P = 0.0004)

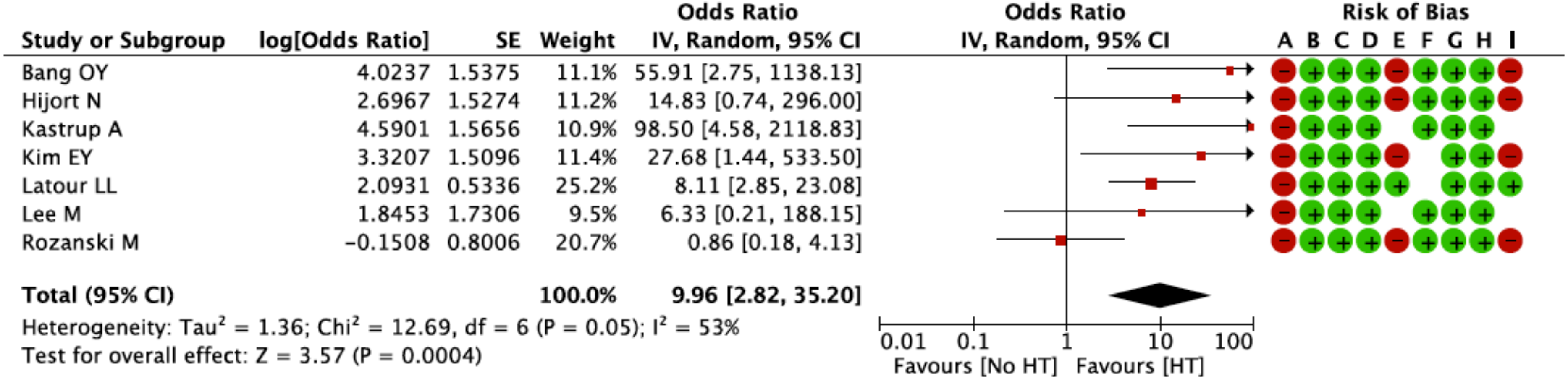

Risk of bias legend

(A) Representativeness
(B) Selection of non–exposed cohort
(C) Ascertainment of exposure
(D) Outcome of interest was not present at the study entry
(E) Comparability
(F) Outcome assessment
(G) Lenght of follow–up
(H) Adequacy of follow–up
(I) Overall
