# Supplementary material for: Blood–Brain Barrier Disruption and Hemorrhagic Transformation in Acute Ischemic Stroke: Systematic Review and Meta-Analysis
Source: Front Neurol. 2021 Jan 21;11:594613. doi: 10.3389/fneur.2020.594613 (PMC7859439; doi:10.3389/fneur.2020.594613)
Supplement: Supplementary file 9 [file Image_3.pdf]

**Supplemental Figure 3.** Relation between BBB (quantitative assessment) and HT in MR studies. BBB, blood–brain barrier; HT, hemorrhagic transformation; MR, magnetic resonance.

| Study or Subgroup | log[Odds Ratio] | SE | Weight | Odds Ratio IV, Random, 95% CI | A | B | C | D | E | F | G | H | I |
|---|---|---|---|---|---|---|---|---|---|---|---|---|---|
| Leigh R | 0.5274 | 0.2269 | 41.3% | 1.69 [1.09, 2.64] | − | + | + | + | + | − | + | + | + |
| Liu HS | 2.8082 | 1.5277 | 26.2% | 16.58 [0.83, 331.11] | − | + | + | + | | + | + | + | |
| Nael K | 3.9398 | 1.0646 | 32.5% | 51.41 [6.38, 414.21] | − | + | + | + | + | − | + | + | + |
| **Total (95% CI)** | | | **100.0%** | **9.33 [0.76, 114.39]** | | | | | | | | | |

Heterogeneity: $Tau^2 = 3.91$; $Chi^2 = 11.74$, df = 2 (P = 0.003); $I^2 = 83\%$

Test for overall effect: Z = 1.75 (P = 0.08)

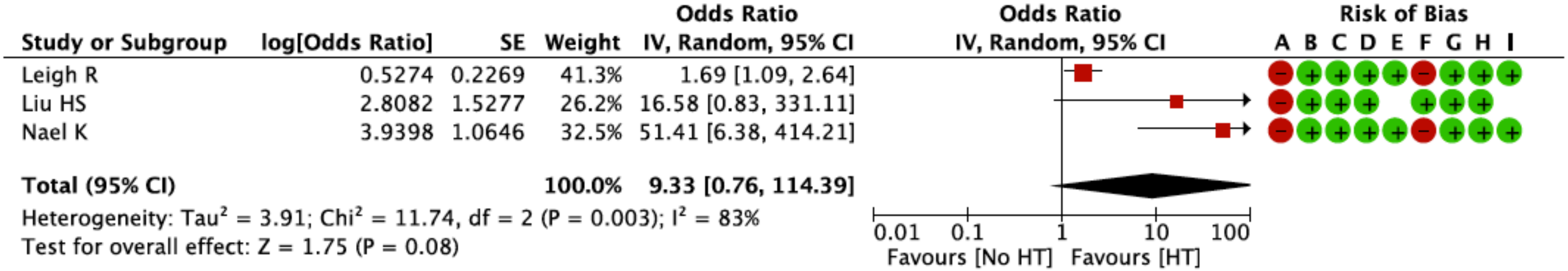


Risk of bias legend
(A) Representativeness
(B) Selection of non-exposed cohort
(C) Ascertainment of exposure
(D) Outcome of interest was not present at the study entry
(E) Comparability
(F) Outcome assessment
(G) Lenght of follow-up
(H) Adequacy of follow-up
(I) Overall
